# Association of Physician Education and Feedback on Hypertension Management With Patient Blood Pressure and Hypertension Control

**DOI:** 10.1001/jamanetworkopen.2019.18625

**Published:** 2020-01-08

**Authors:** Mattias Brunström, Nawi Ng, John Dahlström, Lars H. Lindholm, Göran Lönnberg, Margareta Norberg, Lennarth Nyström, Lars Weinehall, Bo Carlberg

**Affiliations:** 1 Department of Public Health and Clinical Medicine, Umeå University, Umeå, Sweden; 2 Department of Epidemiology and Global Health, Umeå University, Umeå, Sweden; 3 Department of Public Health and Community Medicine, University of Gothenburg, Gothenburg, Sweden

## Abstract

**Importance:**

Elevated systolic blood pressure (SBP) is the most important risk factor for premature death worldwide. However, hypertension detection and control rates continue to be suboptimal.

**Objective:**

To assess the association of education and feedback to primary care physicians with population-level SBP and hypertension control rates.

**Design, Setting, and Participants:**

This pooled series of 108 population-based cohort studies involving 283 079 patients used data from primary care centers in 2 counties (Västerbotten and Södermanland) in Sweden from 2001 to 2009. Participants were individuals aged 18 years or older who had their blood pressure (BP) measured and recorded in either county during the intervention period. All analyses were performed in February 2019.

**Exposures:**

An intervention comprising education and feedback for primary care physicians in Västerbotten County (intervention group) compared with usual care in Södermanland County (control group).

**Main Outcomes and Measures:**

Difference in mean SBP levels between counties and likelihood of hypertension control in the intervention county compared with the control county during 24 months of follow-up.

**Results:**

A total of 136 541 unique individuals (mean [SD] age at inclusion, 64.6 [16.1] years; 57.0% female; mean inclusion BP, 142/82 mm Hg) in the intervention county were compared with 146 538 individuals (mean [SD] age at inclusion, 65.7 [15.9] years; 58.3% female; mean inclusion BP, 144/80 mm Hg) in the control county. Mean SBP difference between counties during follow-up, adjusted for inclusion BP and other covariates, was 1.1 mm Hg (95% CI, 1.0-1.1 mm Hg). Hypertension control improved by 8.4 percentage points, and control was achieved in 37.8% of participants in the intervention county compared with 29.4% in the control county (adjusted odds ratio, 1.30; 95% CI, 1.29-1.31). Differences between counties increased during the intervention period and were more pronounced in participants with higher SBP at inclusion. Results were consistent across all subgroups.

**Conclusions and Relevance:**

This study suggests that SBP levels and hypertension control rates in a county population may be improved by educational approaches directed at physicians and other health care workers. Similar strategies may be adopted to reinforce the implementation of clinical practice guidelines for hypertension management.

## Key Points

**Question:**

Is providing hypertension management education and feedback to primary care physicians associated with reduced systolic blood pressure and improved hypertension control rate in the population?

**Findings:**

In a series of 108 population-based cohort studies involving 283 079 patients, education and feedback provided to primary care physicians were associated with 1.1 mm Hg lower systolic blood pressure in the overall population and 8.4 percentage points of improvement in hypertension control.

**Meaning:**

Findings of this study suggest that education and feedback strategies may reinforce the implementation of clinical practice guidelines for hypertension management.

## Teaser

This cohort study reports on the blood pressure (BP) management program, based on electronic health record system and primary care practitioner education, implemented in primary care facilities in Västerbotten County, Sweden.

## Introduction

Elevated systolic blood pressure (SBP) is the most important risk factor for premature death worldwide.^1^ The effect of agents used to lower blood pressure (BP) on mortality and incident cardiovascular disease (CVD) has been evaluated in numerous randomized clinical trials (RCTs) and is summarized in several systematic reviews and meta-analyses.^2,3,4^ Although some controversy remains regarding the implications of antihypertensive treatment for the nonhypertensive BP range,^5^ all major systematic reviews agree that antihypertensive treatment is associated with reduced risk of death and CVD for an SBP of 140 mm Hg or higher,^2,3,4^ and treatment is recommended for these patients.^6,7^

Despite the overwhelming evidence for the efficacy of treatment, hypertension detection and control rates remain low.^8^ Swedish data suggested that two-thirds of people with hypertension were aware of it, only half received antihypertensive treatment, and two-thirds of those treated reached BP less than 140/90 mm Hg.^9^ Thus, improved detection and control of hypertension could greatly reduce the burden of CVD with the use of inexpensive and readily available drugs.

One factor in suboptimal hypertension control rates is clinical inertia among primary care physicians.^10,11,12^ The Swedish Stroke Prevention Study (SSPS) was developed as a health care intervention to overcome this inertia.^13^ Through an electronic decision support system, lectures on antihypertensive treatment, and feedback on hypertension control, the SSPS aimed to improve the initiation and intensification of BP-lowering treatment by primary care physicians in Västerbotten County, Sweden. In this study, we assessed the association of the intervention with mean SBP levels and hypertension control rates by comparing data from Västerbotten County with data from Södermanland County as the control.

## Methods

The SSPS has been described in detail elsewhere.^13^ The study was approved by the ethics committee at Umeå University. Because the intervention was originally performed as a health care intervention and data for research purposes were collected retrospectively, no informed consent was obtained from individual participants, as approved by the ethics committee. We followed the Strengthening the Reporting of Observational Studies in Epidemiology (STROBE) reporting guidelines.^14^

Briefly, a health care intervention directed at primary care physicians in Västerbotten County was implemented from 2001 to 2009. Primary care in Sweden is tax funded and includes unlimited access as well as capped costs for visits and antihypertensive medications for all permanent residents. The intervention had 3 components. First, in 2001, an electronic decision support system was installed on all physicians’ computers.^15,16^ This system suggested specific antihypertensive agents for each patient according to the 1999 guidelines of the World Health Organization and International Society of Hypertension.^17^ Second, in 2004, 2 investigators gave lectures on antihypertensive treatment at each of the 33 health care centers in Västerbotten County in which the intervention was provided. The lectures focused on (1) the addition of an antihypertensive drug when BP reached 140/90 mm Hg or higher, emphasizing the importance of SBP over diastolic BP (DBP); and (2) the recording of the individualized treatment plan for each patient in the electronic health record. The investigators had interactive discussions with physicians and other staff at each health care center about how to reduce diagnostic and therapeutic barriers. Refresher lectures were given yearly at primary care meetings. Third, from 2007 to 2009, each health care center received feedback on their hypertension control rates; general trends and comparisons between centers were presented at primary care meetings. For comparison, we used data from Södermanland County that continued to provide usual care without any intervention or additional measurements during the study period.

In the present study, we used data from the electronic health record systems of the included counties and from national health registers in Sweden to mimic the eligibility criteria and follow-up of a clinical trial (eMethods in the Supplement).^18^ We created a series of 108 cohort studies, 1 for every month of the study period, from January 1, 2001, to December 31, 2009. Each month, all people in each county aged 18 years or older with BP measurements recorded in a primary care setting were eligible for inclusion. Because most individuals met the eligibility criteria several times, we used the term *individual* to refer to a unique person, whereas the term *participant* referred to a single inclusion of a person.

Participants who had their BP measurements recorded in the intervention county were considered exposed, whereas participants who had their BP measurements recorded in the control county were considered unexposed. Recorded BP levels of people aged 40, 50, or 60 years from both counties were excluded from the analyses to reduce the risk of selection bias owing to the Västerbotten Intervention Programme, a population-based screening and intervention program (including BP recordings) offered to all residents aged 40, 50, or 60 years in the intervention county with approximately 65% participation rates.^19,20^ Explorative analyses found that, in Västerbotten County, the number of measurements in those aged 40, 50, or 60 years compared with other age categories exceeded the expectations by 30% to 50%, with 3 to 8 mm Hg lower mean SBP levels (eTable 1 in the Supplement).

The overall purpose of the SSPS was to assess whether the intervention was associated with reductions in BP and stroke incidence. In this study, we focused on the association between the intervention and BP; stroke and cardiovascular outcomes will be analyzed and reported separately. Co-primary BP outcomes were the differences in mean SBP levels and hypertension control rates between the 2 counties during 24 months of follow-up (until December 31, 2011, for the last cohort). Blood pressure data were extracted from electronic health records using an automated script with greater than 95% sensitivity.^13^ Mean SBP was calculated from all recorded values within 24 months after inclusion. Hypertension control was defined as a mean SBP lower than 140 mm Hg among individuals with previous hypertension diagnosis or an SBP of 140 mm Hg or higher at inclusion. Secondary outcomes were (1) SBP differences between counties stratified by year and by SBP at inclusion and (2) DBP differences between counties overall and stratified by year.

### Statistical Analysis

Linear regression was used to analyze BP differences between counties, and logistic regression analysis was performed to estimate the likelihood of hypertension control. Each monthly cohort was adjusted for the following variables, using the most recent value before inclusion: age (including both linear and quadratic terms in the linear regression model), sex, SBP at inclusion, previous hypertension diagnosis, diabetes, coronary artery disease, stroke, atrial fibrillation, heart failure, marital status, and disposable income per consumption unit (total household income minus taxes, weighted for household composition). To account for multiple inclusions of the same individual, we used a robust variance estimator when cohorts were combined in the overall regression model.^18^ Comorbidity data were collected from the Swedish National Patient Register, and marital status and income data were obtained from Statistics Sweden, both of which were linked to the SSPS data set through the unique Swedish personal identification number.^21^ Educational-level data were also collected from Statistics Sweden but excluded from the primary analyses because the data were available only for participants younger than 75 years, and excluding these older adults would limit the applicability of these analyses. For the secondary outcome, DBP at inclusion replaced SBP at inclusion as covariate.

The association of age, sex, hypertension, diabetes, coronary artery disease, stroke, atrial fibrillation, and heart failure with co-primary outcomes was assessed in subgroup analyses using the fully adjusted model. We performed 3 sets of sensitivity analyses. First, we changed the duration of follow-up from 24 months to 12 months and 36 months to test the association between follow-up and outcome estimates. Second, we excluded SBP at inclusion from the covariate list because this variable may adjust away some of the difference between counties if it were cumulative across cohorts. Third, we included educational level as a covariate, thereby excluding participants older than 75 years. In addition, we calculated E-values, as described by VanderWeele and Ding,^22^ to estimate the strength of association needed for an unmeasured confounder to explain away the observed differences. All analyses were performed in February 2019 using Stata, version 15 (StataCorp LLC).

## Results

In the intervention county, 136 541 unique individuals were analyzed as 743 524 participants in 108 population-based cohorts compared with 146 538 unique individuals analyzed as 841 133 participants in the control county. The mean (SD) age at inclusion was 64.6 (16.1) years in the intervention county and 65.7 (15.9) years in the control county. In the intervention county, 57.0% of participants were female and the mean BP was 142/82 mm Hg, whereas in the control county, 58.3% of participants were female and the mean BP was 144/80 mm Hg. Additional patient characteristics at the time of inclusion are shown in Table 1; the differences between counties were small.

**Table 1.** Patient Characteristics at Time of Inclusion, Stratified by County and Completeness of Follow-up^a^

Variable	Total		With Follow-up Data		Without Follow-up Data	
	Intervention Group	Control Group	Intervention Group	Control Group	Intervention Group	Control Group
Participants (inclusions in cohorts)	743 524 (100)	841 133 (100)	614 053 (82.6)	698 229 (83.0)	129 471 (17.4)	142 904 (16.9)
Unique individuals, No.	136 541	146 538	90 787	97 900	105 597	115 209
Median No. of cohorts per unique individual	3	3	6	6	1	1
Sex						
Female	423 960 (57.0)	490 018 (58.3)	351 237 (57.2)	408 151 (58.5)	72 723 (56.2)	81 867 (57.3)
Male	319 564 (43.0)	351 115 (41.7)	262 816 (42.8)	290 078 (41.5)	56 748 (43.8)	61 037 (42.7)
Age, mean (SD), y	64.6 (16.1)	65.7 (15.9)	66.9 (14.3)	68.0 (14.0)	53.6 (19.6)	54.8 (19.7)
Age group, y						
18-64	325 201 (43.7)	342 552 (40.7)	235 844 (38.4)	246 258 (35.3)	89 357 (69.0)	96 294 (67.4)
65-79	283 623 (38.2)	327 567 (38.9)	258 089 (42.0)	299 621 (42.9)	25 534 (19.7)	27 946 (19.6)
≥80	134 700 (18.1)	171 014 (20.3)	120 120 (19.6)	152 350 (21.8)	14 580 (11.3)	18 664 (13.1)
BP, mean (SD), mm Hg						
SBP	142.1 (20.8)	143.9 (21.4)	144.3 (20.6)	146.2 (21.1)	131.8 (18.4)	132.3 (18.8)
DBP	81.6 (11.2)	80.4 (10.8)	82.2 (11.3)	80.9 (10.9)	78.7 (10.3)	77.7 (9.9)
BP categories, mm Hg						
<120	74 416 (10.0)	71 817 (8.5)	47 712 (7.8)	45 132 (6.5)	26 704 (20.6)	26 685 (18.7)
120-129	105 676 (14.2)	106 938 (12.7)	75 758 (12.3)	74 547 (10.7)	29 918 (23.1)	32 391 (22.7)
130-139	137 793 (18.5)	136 889 (16.3)	110 387 (18.0)	109 120 (15.6)	27 406 (21.2)	27 769 (19.4)
140-149	157 489 (21.2)	183 885 (21.9)	134 712 (21.9)	155 846 (22.3)	22 777 (17.6)	28 039 (19.6)
150-159	103 715 (14.0)	124 155 (14.8)	92 956 (15.1)	110 833 (15.9)	10 759 (8.3)	13 322 (9.3)
160-169	80 549 (10.8)	104 102 (12.4)	73 851 (12.0)	95 615 (13.7)	6698 (5.2)	8487 (5.9)
170-179	39 707 (5.3)	50 939 (6.1)	36 989 (6.0)	47 813 (6.9)	2718 (2.1)	3126 (2.2)
≥180	44 179 (5.9)	62 408 (7.4)	41 688 (6.8)	59 323 (8.5)	2491 (1.9)	3085 (2.2)
Comorbidities						
Diabetes	125 738 (16.9)	165 438 (19.7)	119 564 (19.5)	157 831 (22.6)	6174 (4.8)	7607 (5.3)
Coronary artery disease	101 247 (13.6)	89 538 (10.6)	91 797 (15.0)	81 064 (11.6)	9450 (7.3)	8474 (5.9)
Previous stroke	45 516 (6.1)	45 455 (5.4)	40 608 (6.6)	40 654 (5.8)	4908 (3.8)	4801 (3.4)
Heart failure	34 294 (4.6)	31 352 (3.7)	30 049 (4.9)	26 924 (3.9)	4245 (3.3)	4428 (3.1)
Atrial fibrillation	48 031 (6.5)	45 773 (5.4)	42 972 (7.0)	40 917 (5.9)	5059 (3.9)	4856 (3.4)
Disposable income						
Quintile 1	147 231 (19.8)	168 994 (20.1)	120 278 (19.6)	137 352 (19.7)	26 953 (20.9)	31 642 (22.3)
Quintile 2	146 670 (19.8)	169 568 (20.2)	124 485 (20.3)	145 132 (20.8)	22 185 (17.2)	24 436 (17.2)
Quintile 3	147 909 (19.9)	168 301 (20.1)	123 849 (20.2)	142 315 (20.4)	24 060 (18.7)	25 986 (18.3)
Quintile 4	152 823 (20.6)	163 403 (19.5)	125 016 (20.4)	134 854 (19.4)	27 807 (21.6)	28 549 (20.1)
Quintile 5	147 445 (19.9)	168 777 (20.1)	119 600 (19.5)	137 191 (19.7)	27 845 (21.6)	31 586 (22.2)
Civil status						
Unmarried	134 047 (18.1)	115 462 (13.8)	90 677 (14.8)	76 071 (10.9)	43 370 (33.7)	39 391 (27.7)
Married	383 364 (51.7)	446 586 (53.2)	325 464 (53.1)	379 781 (54.5)	57 900 (45.0)	66 805 (47.0)
Divorced	84 331 (11.4)	116 983 (14.0)	70 594 (11.5)	97 077 (13.9)	13 737 (10.7)	19 906 (14.0)
Widowed	140 126 (18.9)	159 740 (19.0)	126 384 (20.6)	143 744 (20.6)	13 742 (10.7)	15 996 (11.3)
Educational level^b^						
Elementary school	159 382 (30.3)	218 097 (38.1)	136 673 (32.6)	185 902 (40.6)	22 709 (21.2)	32 195 (28.0)
Secondary school	249 503 (47.4)	251 135 (43.9)	198 249 (47.2)	194 836 (42.6)	51 254 (47.9)	56 299 (48.9)
University degree	117 790 (22.4)	103 470 (18.1)	84 833 (20.2)	76 823 (16.8)	32 957 (30.8)	26 647 (23.1)

Abbreviations: BP, blood pressure; DBP, diastolic blood pressure; SBP, systolic blood pressure.

^a^ Values given as number (percentage) unless otherwise indicated.

^b^ Available only for people younger than 75 years.

The 24-month follow-up BP data were available for 614 053 participants (82.6%) in the intervention county and for 698 229 (83.0%) in the control county. Patient characteristics differed between participants with and those without follow-up data. Participants without follow-up data were younger, had lower BP, had fewer comorbidities, were more likely to be unmarried, and were more likely to be in either the lowest or highest quintile for disposable income. The mean number of BP recordings for participants with follow-up data during 24 months were 4.2 for the intervention county and 4.7 for the control county.

A total of 562 330 participants (75.6%) in the intervention county and 631 064 participants (75.0%) in the control county had a previous diagnosis of hypertension or an SBP of 140 mm Hg or higher at the time of inclusion and were thus categorized as having hypertension. Follow-up data were available for 503 573 participants (89.6%) in the intervention county and 565 820 participants (89.7%) in the control county.

### Primary Outcomes

**Figure 1.**
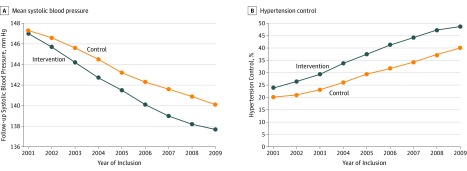
Follow-up Systolic Blood Pressure and Hypertension Control by Year of Inclusion

Follow-up SBP decreased in both counties during the study period, with more pronounced changes in the intervention county (Figure 1A; Table 2). The unadjusted difference in follow-up SBP was −0.3 mm Hg (95% CI, −0.5 to −0.1 mm Hg) for cohorts included in the first year, increasing progressively to −2.7 mm Hg (95% CI, −2.8 to −2.5 mm Hg) for cohorts included in 2008. In the fully adjusted model, differences between counties decreased compared with the unadjusted model; the mean difference between counties was 1.1 mm Hg (95% CI, 1.0-1.1) for all years combined, ranging from 0.7 mm Hg to 1.3 mm Hg for different years of inclusion.

**Table 2.** Mean Systolic Blood Pressure During Follow-up by Year of Inclusion

Variable	2001	2002	2003	2004	2005	2006	2007	2008	2009
No. of participants									
Intervention group	65 706	68 596	70 075	75 604	81 987	93 437	98 320	95 802	93 997
Control group	87 394	87 739	92 240	93 544	91 720	95 079	99 663	98 305	95 449
Mean SBP at inclusion, mm Hg									
Intervention group	147.7	146.3	145.2	144.0	142.4	141.2	139.6	138.8	138.1
Control group	147.2	146.6	145.9	144.9	143.7	142.9	142.3	141.5	140.6
Mean follow-up SBP, mm Hg									
Intervention group	147.0	145.7	144.2	142.7	141.5	140.1	139.0	138.2	137.7
Control group	147.3	146.6	145.6	144.5	143.2	142.3	141.6	140.9	140.1
SBP difference between counties during follow-up, mm Hg (95% CI)									
Unadjusted	–0.3 (–0.1 to –0.5)	–0.9 (–0.7 to –1.1)	–1.3 (–1.2 to –1.5)	–1.7 (–1.5 to –1.9)	–1.7 (–1.5 to –1.9)	–2.2 (–2.0 to –2.3)	–2.6 (–2.4 to –2.7)	–2.7 (–2.5 to –2.8)	–2.4 (–2.3 to –2.6)
Fully adjusted^a^	–0.7 (–0.6 to –0.9)	–0.8 (–0.7 to –1.0)	–1.1 (–0.9 to –1.2)	–1.3 (–1.2 to –1.4)	–1.1 (–1.0 to –1.2)	–1.2 (–1.1 to –1.3)	–1.2 (–1.1 to –1.3)	–1.3 (–1.1 to –1.4)	–1.2 (–1.1 to –1.3)

Abbreviation: SBP, systolic blood pressure.

^a^ Analyses were adjusted for age (linear and nonlinear), sex, SBP at inclusion, previous hypertension diagnosis, diabetes, coronary artery disease, stroke, atrial fibrillation, heart failure, marital status, and household income per consumption unit.

Hypertension control improved by 8.4 percentage points, and control was achieved in 190 406 participants (37.8%) in the intervention county compared with 166 560 participants (29.4%) in the control county for all years combined. Hypertension control rates increased during the study period from just above 20% for participants included in 2001 to almost 50% for participants included in 2009. Differences between counties increased progressively, with the largest absolute difference between counties for cohorts included in 2008 (47.2% vs 37.3%) (Figure 1B; Table 3). The crude odds ratio for treatment control was 1.46 (95% CI, 1.45-1.47), whereas the fully adjusted odds ratio was 1.30 (95% CI, 1.29-1.31) for all years combined. Odds ratios for hypertension control by year of inclusion are shown in Figure 1B.

**Table 3.** Hypertension Control During Follow-up by Year of Inclusion

Variable	2001	2002	2003	2004	2005	2006	2007	2008	2009
No. of participants									
Intervention	52 276	54 139	54 655	58 629	62 673	70 136	71 874	69 928	68 020
Control	65 932	66 315	69 907	70 592	68 593	70 591	74 219	73 578	71 337
Hypertension control, %									
Intervention	23.7	26.4	29.3	33.7	37.4	41.1	44.1	47.2	48.6
Control	20.1	21.0	23.1	26.0	29.4	31.6	34.2	37.3	40.0
OR for hypertension control (95 % CI)									
Unadjusted	1.23 (1.20 to 1.27)	1.35 (1.31 to 1.39)	1.38 (1.34 to 1.42)	1.44 (1.41 to 1.48)	1.44 (1.40 to 1.47)	1.51 (1.48 to 1.55)	1.52 (1.48 to 1.55)	1.50 (1.47 to 1.54)	1.41 (1.38 to 1.45)
Fully adjusted^a^	1.15 (1.11 to 1.18)	1.23 (1.19 to 1.26)	1.26 (1.23 to 1.30)	1.31 (1.28 to 1.35)	1.31 (1.27 to 1.35)	1.37 (1.34 to 1.41)	1.35 (1.31 to 1.38	1.35 (1.32 to 1.39)	1.29 (1.26 to 1.32)

Abbreviation: OR, odds ratio.

^a^ Analyses for hypertension control include only participants with hypertension diagnosis or systolic blood pressure of 140 mm Hg or higher at the time of inclusion. Adjusted for age, sex, systolic blood pressure at inclusion, previous hypertension diagnosis, diabetes, coronary artery disease, stroke, atrial fibrillation, heart failure, marital status, and household income per consumption unit.

### Secondary Outcomes

Differences in follow-up SBP levels between counties were more pronounced in the highest SBP categories (eTable 2 in the Supplement). The general pattern, with increased differences during the intervention period, was robust across BP levels. The DBP levels decreased in both counties during the intervention period but less so compared with SBP levels (eTable 3 in the Supplement). The decrease was more pronounced in the control county compared with the intervention county, with a difference of 0.5 mm Hg between counties in the fully adjusted model for all years combined.

### Subgroup Analyses

**Figure 2.**
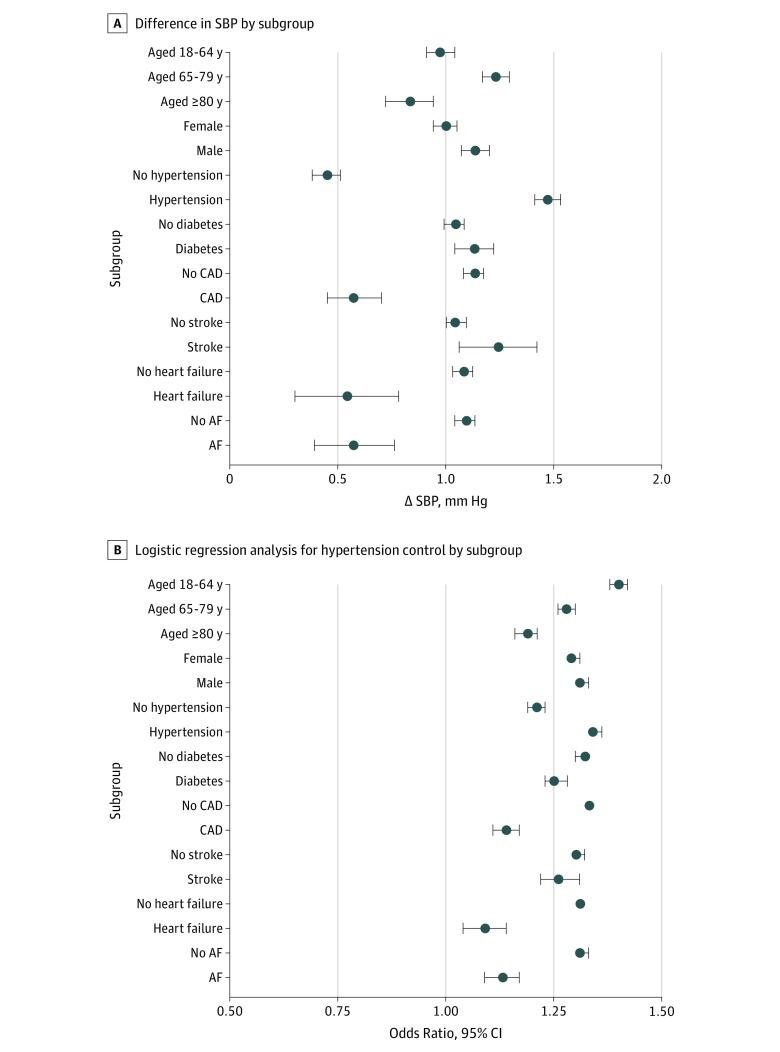
Systolic Blood Pressure (SBP) Difference and Odds Ratio for Hypertension Control by Subgroups Analyses for hypertension control included only participants with hypertension diagnosis or systolic blood pressure of 140 mm Hg or higher at the time of inclusion. AF indicates atrial fibrillation; CAD, coronary artery disease.

Subgroup analyses consistently found lower SBP (Figure 2A) and increased likelihood of hypertension control (Figure 2B) in the intervention county compared with the control county during follow-up. Differences between counties were larger for participants with hypertension compared with those without hypertension, whereas other comorbidities were associated with smaller differences. As shown in eTable 4 in the Supplement, mean BP levels were lower in patients with comorbidities (other than diabetes), which may be a factor in the smaller difference between counties observed for participants with comorbidities. For diabetes, much attention was given to risk factor control nationally during the intervention period, which may be associated with the diminished differences between counties. Age group analyses were inconsistent, with the largest mean SBP difference in the 65-to-79-year age group, but the largest difference in hypertension control rates was in the 18-to-64-year age group, probably reflecting the lower prevalence of hypertension in the youngest age group.

### Sensitivity Analyses

Excluding SBP at inclusion from the fully adjusted model increased the differences between counties to levels closer to the unadjusted model than the fully adjusted model (eTable 5 in the Supplement). Including educational level as a covariate (thereby excluding participants aged >75 years) did not alter the direction or magnitude of the findings; neither did shifting follow-up duration to 12 or 36 months. E-values were 1.31 for the mean SBP difference between counties and 1.54 for hypertension control, indicating that any unmeasured confounder would need to be associated with both county and hypertension by a magnitude of at least 1.31 on the risk ratio scale or with county and SBP by a magnitude of 1.54 to explain away the findings (eResults in the Supplement).

## Discussion

In this article, we report BP outcome from the SSPS. During the intervention period from 2001 to 2009, the mean SBP level decreased more in the intervention county compared with the control county. Hypertension control rates increased substantially in both counties but more so in the intervention county, with 30% higher odds for BP control in the fully adjusted model. Differences between counties increased during the intervention period, probably reflecting the progressive nature of the intervention. These findings suggest that it is possible and feasible to lower SBP and improve hypertension control rates in the population through interventions directed at primary care.

### Comparison With Previous Studies

To our knowledge, this study is the first to evaluate the association between an educational intervention for primary care physicians and population-based risk factor control. Because the intervention was provided in a real-world setting with usual care BP recordings for inclusion and follow-up, the results should be highly applicable to clinical practice. This applicability is a strength of this study over RCTs, whose generalizability often has been questioned owing to their narrow eligibility criteria and rigorous follow-up procedures, which are lacking in reality.^23,24^ In this study, all adults with BP recordings were included without restrictions on age, comorbidities, and concurrent treatment. Thus, the BP measurements reported here are representative of the overall population receiving primary care, with two-thirds of participants being older adults and more than 20% being 80 years or older by the time of inclusion.

The overall difference in hypertension control rate reported here was similar to the effect size reported in RCTs of interventions to reduce clinical inertia.^25^ The number of unique individuals included in the present analysis exceeded by more than 10-fold the number of participants included in all RCTs combined.^25^ Although this study’s findings are less reliable in demonstrating causal inference compared with results of RCTs, the large number of included individuals allowed us to explore interactions between covariates in a detailed way with sustained statistical power to detect small differences between counties.

This study offers insights that add to those in previous reports from large-scale hypertension initiatives, such as that of Kaiser Permanente Northern California^26^ and the Canadian Hypertension Education Program.^27^ The hypertension programs of Kaiser Permanente Northern California is an integrated health care system with both outpatient and inpatient care, and the Canadian Hypertension Education Program comprehensively address all aspects of hypertension management; in contrast, the intervention assessed in the present study was simple, was introduced in an existing primary care system, and required minimal resources. From a methodological perspective, the comparisons of the hypertension control rates between the Kaiser Permanente Northern California program and regional and national initiatives may be confounded by other aspects of care as well as demographic and socioeconomic factors.^26^ The comparison between 2 counties in this study was adjusted for several individual-level comorbidities as well as demographic and socioeconomic factors.

Analyzing data as a series of 108 cohort studies, with the potential to include individuals each time they have their BP recorded, had 2 main advantages compared with the traditional approach of simply comparing county residents or including people only once.^18,28^ First, each time people had their BP measured in the intervention county, they were subject to the intervention. Starting follow-up at the time of BP recording rather than analyzing county residents by year minimized the risk of selection bias occurring from the time of the intervention to the start of follow-up, sometimes referred to as immortal-time bias.^29^ Second, if time trends were present, as expected with a stepwise intervention, including people only when they were first eligible would skew estimates toward the beginning of the study period. Including individuals every time they fulfill the eligibility criteria, however, created a study base that is balanced across the study period.

### Implications for Clinical Practice

In a recent systematic review of RCTs, antihypertensive treatment was associated with a 4.6–mm Hg lower SBP and 12% lower risk for CVD if the baseline SBP was 140 to 159 mm Hg,^4^ corresponding to the mean SBP at inclusion in the present study. During the late phase of the intervention, the absolute difference in follow-up BP measurement between counties was about half of that observed in the previous meta-analysis, diminishing to about one-fourth when analyses were adjusted for SBP at inclusion. Such a difference would translate into a relative risk reduction for CVD between 3% and 6% if trial results were applicable to general practice. According to nationwide Swedish data, two-thirds of all patients who experience a stroke (approximately 14 000 yearly) have a history of hypertension.^30^ Based on these numbers, an intervention similar to the one assessed here should be associated with 400 to 800 fewer strokes per year in Sweden. For myocardial infarction, the corresponding estimate would be 300 to 600 patients,^31^ resulting in approximately 700 to 1400 fewer cardiovascular events in Sweden, which has approximately 10 million inhabitants.

The education and feedback strategy assessed in the present cohort study could possibly be extended to other preventive efforts, such as lipid-lowering treatment or diabetes management. Given the limited resources and an increasing number of chronic conditions handled in primary care, feedback and therapeutic suggestions may lower treatment thresholds, thereby reducing cardiovascular risk and improving longevity. Although similar interventions across a broad range of conditions may attenuate its effectiveness, the intervention could be applied to areas of special interest, ideally chosen after careful analyses of risk factors and clinical outcomes in the population.

### Limitations

This study has some limitations. The aim of this study was to establish the association between the intervention and BP control, but the findings should be regarded as observational because of the lack of randomization. Although we were able to include the most important risk factors for hypertension (age, sex, and diabetes) as well as previous CVD and socioeconomic factors as covariates in these analyses, data on other risk factors (such as obesity and physical activity) were not available. Such differences could possibly confound the observed trends for BP at inclusion, but they were less likely to affect BP level change during follow-up because adjustment for SBP levels at inclusion indirectly adjusted for other factors affecting BP levels before inclusion. Thus, for an unmeasured confounder to alter the results, it would have to appear during the 24 months after inclusion into each cohort. The calculated E-value for hypertension control rate further suggested that such an unmeasured confounder would need to be 54% more common in the control county and increase the likelihood of hypertension control by 54% to explain away the findings. Such a large difference between counties for an unmeasured confounder seems unlikely given the observed similarities for other parameters.

Special considerations had to be made to minimize the influence of the Västerbotten Intervention Programme on this study’s results.^19^ The Västerbotten Intervention Programme invites all Västerbotten County residents aged 40, 50, or 60 years to undergo an extensive health check, including BP measurement. Thus, inclusion of individuals aged 40, 50, or 60 years in the present analyses would have introduced selection bias, because participants in the Västerbotten Intervention Programme were likely healthier compared with those who had their BP measured in usual care. This theory was supported by a vast increase in the number of measurements and substantially lower mean BP values for recordings among participants aged 40, 50, or 60 years in the intervention county compared with adjacent ages in the intervention county or similar ages in the control county (eTable 1 in the Supplement). Because we had no indicator in the SSPS database about which records were derived from the Västerbotten Intervention Programme, we excluded all records from participants aged 40, 50, or 60 years from both counties. This exclusion will minimize selection bias from the Västerbotten Intervention Programme, but spillover effects are possible, although unlikely, given that the program is offered only 3 times, in 10-year intervals, during a person’s lifespan and does not focus on hypertension specifically.

Another possible source of bias may occur if the SSPS intervention made primary care physicians more prone to measure and record BP through increased awareness of BP as a risk factor. As a result, observed BP levels in the intervention county would be lower compared with the control county owing to the inclusion of healthier participants rather than lower BP measurements in similar participants. To account for this possibility, we used SBP level at inclusion as a covariate in the fully adjusted model, noting that, if the progressively lower inclusion BP levels in the intervention county were from a previous intervention among individuals who later appeared as new participants, then we would underestimate the difference between counties associated with the intervention.

## Conclusions

The results of this cohort study show that, during the study period from 2001 to 2009, BP levels decreased more in Västerbotten County, where the intervention was implemented to reduce clinical inertia among primary care physicians, compared with Södermanland County, where usual care continued. These results should be interpreted with caution because of the observational nature of our analyses, but the findings suggest that BP levels and the hypertension control rate in a population could potentially be improved by county-level strategies directed at physicians and other health care workers.

## Notes

Supplement. **eMethods.** Underlying Principle and Example of Inclusion and Follow-up for an Individual **eTable 1.** Number of Measurements and Average Blood Pressure Level by Age, Including 40-, 50-, and 60-Year-Olds **eTable 2.** Systolic Blood Pressure Difference Between Intervention and Control County by Year of Inclusion and Systolic Blood Pressure Category at Inclusion **eTable 3.** Diastolic Blood Pressure Difference Between Intervention and Control County by Year of Inclusion **eTable 4.** Mean Inclusion Blood Pressure by County and Co-morbidity **eTable 5.** Sensitivity Analyses **eResults.** E-values for Co-primary Outcomes
