# Supplementary material for: Association of Physician Education and Feedback on Hypertension Management With Patient Blood Pressure and Hypertension Control
Source: JAMA Netw Open. 2020 Jan 8;3(1):e1918625. doi: 10.1001/jamanetworkopen.2019.18625 (PMC6991247; doi:10.1001/jamanetworkopen.2019.18625)
Supplement: Supplement. — eMethods. Underlying Principle and Example of Inclusion and Follow-up for an Individual eTable 1. Number of Measurements and Average Blood Pressure Level by Age, Including 40-, 50-, and 60-Year-Olds eTable 2. Systolic Blood Pressure Difference Between Intervention and Control County by Year of Inclusion and Systolic Blood Pressure Category at Inclusion eTable 3. Diastolic Blood Pressure Difference Between Intervention and Control County by Year of Inclusion eTable 4. Mean Inclusion Blood Pressure by County and Co-morbidity eTable 5. Sensitivity Analyses eResults. E-values for Co-primary Outcomes [file jamanetwopen-3-e1918625-s001.pdf]

Supplementary Online Content

Brunström M, Ng N, Dahlström J, et al. Association of physician education and feedback on hypertension management with patient blood pressure and hypertension control. *JAMA Netw Open.* 2020;3(1):e1918625.
doi:10.1001/jamanetworkopen.2019.18625

eMethods. Underlying Principle and Example of Inclusion and Follow-up for an Individual

eTable 1. Number of Measurements and Average Blood Pressure Level by Age, Including 40-, 50-, and 60-Year-Olds

eTable 2. Systolic Blood Pressure Difference Between Intervention and Control County by Year of Inclusion and Systolic Blood Pressure Category at Inclusion

eTable 3. Diastolic Blood Pressure Difference Between Intervention and Control County by Year of Inclusion

eTable 4. Mean Inclusion Blood Pressure by County and Co-morbidity

eTable 5. Sensitivity Analyses

eResults. E-values for Co-primary Outcomes

This supplementary material has been provided by the authors to give readers additional information about their work.

eMethods. Underlying Principle and Example of Inclusion and Follow-up for an Individual

Underlying principle
We have used the emulated target-trial principle to analyze data in this study. The basic principle with this method is to define eligibility criteria in the same way as for a randomized controlled trial, and to include individuals whenever they meet these inclusion criteria. This results in multiple inclusions of the same individual, which might be counterintuitive from a traditional epidemiological point of view. However, the aim with this method is not to describe the population, but to estimate the causal effect of an intervention, and therefore minimizing bias is prioritized over representativeness.

Example of inclusion and follow-up for an individual
The figure below aims to depict how one individual may be included in the study. The first blood pressure for individual 1 is recorded in January 2001 and he is therefore included in cohort 1. The consecutive 24 months serve as follow-up; the mean of the second, third and fourth recording will be his follow-up blood pressure. Individual 1 has no recordings during February 2001 and is therefore not included in cohort 2 (or 3 or 4). The next measurement is in May 2001; individual 1 is included in cohort 5 and recording three, four and five are included in the follow-up. Same principle goes for the third and fourth recording. For the fifth recording, in March 2003, individual 1 fulfil the eligibility criteria, but does not have any follow-up data and can therefore not be included in the analyses.

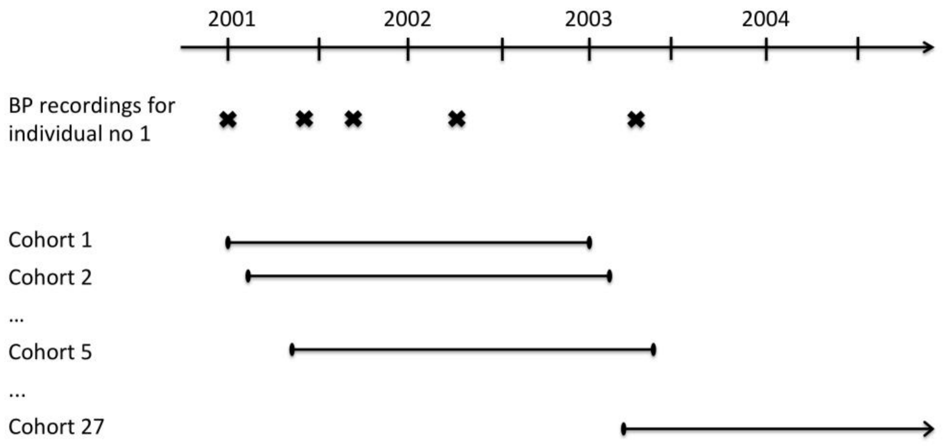

©2020 Brunström M et al. *JAMA Network Open.*

eTable 1. Number of Measurements and Average Blood Pressure Level by Age, Including 40-, 50-, and 60-Year-Olds

Age at measurement (years)									
	39	**40**	41	49	**50**	51	59	**60**	61
No. intervent (n)	14 604	**25 129**	15 243	38 222	**61 463**	44 243	77 936	**108 101**	75 310
No. control (n)	13 236	**14 133**	14 844	36 404	**38 432**	41 692	85 275	**87 801**	95 326
SBT intervent (mm Hg)	129.8	**121.9**	130.8	137.5	**130.6**	138.1	141.9	**138.6**	142.5
SBT control (mm Hg)	128.5	**129.0**	129.8	137.8	**138.4**	139.2	144.2	**144.2**	144.6
DBT intervent (mm Hg)	83.1	**76.8**	83.4	86.4	**81.9**	86.5	85.4	**83.3**	84.7
DBT control (mm Hg)	80.1	**80.4**	80.7	84.0	**84.2**	84.3	83.8	**83.4**	83.3

Absolute numbers of recordings, and unadjusted mean systolic blood pressure (SBT) and diastolic blood pressure (DBT) according to age at recording.

©2020 Brunström M et al. *JAMA Network Open.*

eTable 2. Systolic Blood Pressure Difference Between Intervention and Control County by Year of Inclusion and Systolic Blood Pressure Category at Inclusion

	Inclusion year								
SBP category at inclusion (mm Hg)	2001	2002	2003	2004	2005	2006	2007	2008	2009
< 120	0.0 (-0.6 to 0.7)	0.4 (-0.2 to 1.0)	-0.3 (-0.8 to 0.3)	-0.8 (-1.3 to -0.2)	-0.4 (-1.0 to 0.1)	-0.5 (-1.0 to -0.1)	0.1 (-0.3 to 0.6)	0.0 (-0.4 to 0.4)	-0.4 (-0.9 to -0.0)
120-129	0.4 (-0.1 to 0.9)	-0.2 (-0.7 to 0.3)	-0.5 (-0.9 to -0.1)	-0.4 (-0.8 to -0.0)	-0.6 (-0.9 to -0.2)	-0.7 (-1.0 to -0.4)	-0.4 (-0.7 to -0.1)	-0.7 (-1.1 to -0.4)	-0.8 (-1.1 to -0.5)
130-139	-0.6 (-1.0 to -0.2)	-0.4 (-0.8 to -0.0)	-1.0 (-1.4 to -0.7)	-0.8 (-1.1 to -0.5)	-0.9 (-1.2 to -0.6)	-1.1 (-1.4 to -0.8)	-0.8 (-1.0 to -0.5)	-1.1 (-1.4 to -0.8)	-1.1 (-1.4 to -0.9)
140-149	-0.8 (-1.1 to -0.5)	-0.6 (-0.8 to -0.3)	-0.9 (-1.1 to -0.6)	-1.0 (-1.3 to -0.8)	-0.9 (-1.2 to -0.7)	-1.3 (-1.5 to -1.1)	-1.1 (-1.3 to -0.9)	-1.3 (-1.6 to -1.1)	-1.4 (-1.6 to -1.1)
150-159	-0.6 (-0.9 to -0.3)	-1.2 (-1.5 to -0.9)	-1.1 (-1.4 to -0.8)	-1.8 (-2.1 to -1.5)	-1.4 (-1.8 to -1.1)	-1.4 (-1.8 to -1.1)	-1.7 (-2.0 to -1.4)	-1.7 (-2.0 to -1.4)	-1.3 (-1.6 to -1.0)
160-169	-1.4 (-1.7 to -1.0)	-1.3 (-1.6 to -0.9)	-1.1 (-1.4 to -0.7)	-2.0 (-2.3 to -1.6)	-1.6 (-2.0 to -1.3)	-1.7 (-2.0 to -1.3)	-1.6 (-2.0 to -1.3)	-1.7 (-2.1 to -1.3)	-1.6 (-2.0 to -1.2)
170-179	-1.5 (-2.0 to -1.0)	-1.7 (-2.2 to -1.2)	-2.5 (-3.0 to -2.0)	-2.0 (-2.6 to -1.5)	-2.0 (-2.6 to -1.5)	-1.4 (-1.9 to -0.8)	-2.7 (-3.2 to -2.1)	-1.6 (-2.2 to -1.1)	-2.1 (-2.7 to -1.4)
≥180	-1.4 (-1.9 to -0.9)	-2.0 (-2.5 to -1.5)	-2.0 (-2.5 to -1.5)	-2.2 (-2.7 to -1.7)	-1.6 (-2.1 to -1.0)	-1.8 (-2.4 to -1.2)	-2.7 (-3.3 to -2.1)	-2.6 (-3.2 to -1.9)	-2.0 (-2.7 to -1.3)

Systolic blood pressure difference between intervention and control county during 24 months follow-up by inclusion year and systolic blood pressure category. Values are in mm Hg (95 % confidence intervals), derived from the fully adjusted linear regression model including the following covariates: age (linear), age (non-linear), sex, systolic blood pressure at inclusion, previous hypertension diagnosis, diabetes, coronary artery disease, stroke, atrial fibrillation, heart failure, marital status, and household income per consumption unit.

©2020 Brunström M et al. *JAMA Network Open*

eTable 3. Diastolic Blood Pressure Difference Between Intervention and Control County by Year of Inclusion

**No of participants**	2001	2002	2003	2004	2005	2006	2007	2008	2009
- Intervention	65 706	68 596	70 075	75 604	81 987	93 437	98 320	95 802	93 997
- Control	87 394	87 739	92 240	93 544	91 720	95 079	99 663	98 305	95 449
**Mean diastolic blood pressure at inclusion**									
- Intervention	83.3	82.6	82.1	81.8	81.4	81.4	81.0	80.8	80.8
- Control	81.5	81.0	80.9	80.5	80.2	80.1	79.9	79.8	79.7
**Mean follow-up diastolic blood pressure**									
- Intervention	82.3	81.7	81.2	80.9	80.7	80.5	80.3	80.1	80.1
- Control	80.8	80.3	80.1	79.8	79.4	79.2	79.0	79.0	79.0
**Follow-up diastolic blood pressure difference between counties**									
- Unadjusted	1.6 (1.5 to 1.7)	1.4 (1.3 to 1.5)	1.2 (1.1 to 1.3)	1.1 (1.0 to 1.2)	1.3 (1.2 to 1.4)	1.3 (1.3 to 1.4)	1.2 (1.1 to 1.3)	1.0 (1.0 to 1.1)	1.2 (1.1 to 1.3)
- Fully adjusted	0.4 (0.3 to 0.5)	0.4 (0.3 to 0.5)	0.4 (0.3 to 0.4)	0.3 (0.2 to 0.3)	0.5 (0.5 to 0.6)	0.7 (0.6 to 0.7)	0.7 (0.6 to 0.8)	0.6 (0.5 to 0.7)	0.6 (0.6 to 0.7)

Diastolic blood pressure level at inclusion and during 24 months follow-up, and diastolic blood pressure difference between intervention and control county, by year of inclusion. Values are in mmHg (95 % confidence intervals). The fully adjusted model included the following covariates: age (linear), age (non-linear), sex, diastolic blood pressure at inclusion, previous hypertension diagnosis, diabetes, coronary artery disease, stroke, atrial fibrillation, heart failure, marital status, and household income per consumption unit.

©2020 Brunström M et al. *JAMA Network Open.*

eTable 4. Mean Inclusion Blood Pressure by County and Co-morbidity

	Intervention County	Control county
**Hypertension**	146.8/82.9	150.2/82.0
**No Hypertension**	136.4/80.0	137.5/78.7
**Diabetes**	142.9/79.4	144.9/78.6
**No Diabetes**	142.0/82.1	143.6/80.8
**CAD**	140.8/77.8	141.6/77.0
**No CAD**	142.4/82.2	144.1/80.8
**Stroke**	141.2/78.3	143.9/78.2
**No stroke**	142.2/81.8	143.9/80.5
**Heart failure**	135.2/74.9	136.4/75.1
**No heart failure**	142.5/81.9	144.2/80.6
**AF**	139.7/78.4	140.7/77.9
**No AF**	142.3/81.8	144.1/80.5

Unadjusted blood pressure at inclusion by county and co-morbidity. CAD = coronary artery disease. AF = atrial fibrillation.

©2020 Brunström M et al. *JAMA Network Open.*

eTable 5. Sensitivity Analyses

	Inclusion year									
**Systolic blood pressure difference between counties**	2001	2002	2003	2004	2005	2006	2007	2008	2009	All years combined
Unadjusted	-0.3 (-0.5 to -0.1)	-0.9 (-1.1 to -0.7)	-1.3 (-1.5 to -1.2)	-1.7 (-1.9 to -1.5)	-1.7 (-1.9 to -1.5)	-2.2 (-2.3 to -2.0)	-2.6 (-2.7 to -2.4)	-2.7 (-2.8 to -2.5)	-2.4 (-2.6 to -2.3)	-2.1 (-2.2 to -2.0)
Fully adjusted	-0.7 (-0.9 to -0.6)	-0.8 (-1.0 to -0.7)	-1.1 (-1.2 to -0.9)	-1.3 (-1.4 to -1.2)	-1.1 (-1.2 to -1.0)	-1.2 (-1.3 to -1.1)	-1.2 (-1.3 to -1.1)	-1.3 (-1.4 to -1.1)	-1.2 (-1.3 to -1.1)	-1.1 (-1.1 to -1.0)
- Follow-up duration 12 months	-0.7 (-0.8 to -0.5)	-0.9 (-1.1 to -0.8)	-0.9 (-1.1 to -0.8)	-1.2 (-1.3 to -1.1)	-1.1 (-1.2 to -1.0)	-1.2 (-1.3 to -1.1)	-1.3 (-1.5 to -1.2)	-1.2 (-1.4 to -1.1)	-1.1 (-1.3 to -1.0)	-1.0 (-1.1 to -1.0)
- Follow-up duration 36 months	-0.8 (-0.9 to -0.6)	-0.9 (-1.0 to -0.8)	-1.2 (-1.3 to -1.0)	-1.4 (-1.5 to -1.3)	-1.2 (-1.3 to -1.1)	-1.2 (-1.3 to -1.1)	-1.2 (-1.3 to -1.1)	-1.3 (-1.4 to -1.2)	-1.2 (-1.3 to -1.1)	-1.1 (-1.1 to -1.1)
- Excluding SBP at inclusion as covariate	-0.9 (-1.0 to -0.7)	-1.2 (-1.4 to -1.0)	-1.5 (-1.6 to -1.3)	-1.8 (-1.9 to -1.6)	-1.7 (-1.9 to -1.6)	-1.9 (-2.1 to -1.8)	-2.1 (-2.2 to -2.0)	-2.1 (-2.3 to -2.0)	-2.0 (-2.1 to -1.8)	-1.7 (-1.7 to -1.6)
- Including educational level as covariate	-0.6 (-0.8 to -0.5)	-0.7 (-0.8 to -0.5)	-1.1 (-1.2 to -0.9)	-1.3 (-1.4 to -1.1)	-1.2 (-1.3 to -1.0)	-1.2 (-1.3 to -1.1)	-1.2 (-1.4 to -1.1)	-1.4 (-1.5 to -1.2)	-1.4 (-1.5 to -1.2)	-1.1 (-1.1 to -1.0)

Systolic blood pressure difference between intervention and control county. Values are in mmHg (95 % confidence intervals). The fully adjusted model includes the following covariates: age (linear), age (non-linear), sex, systolic blood pressure at inclusion, previous hypertension diagnosis, diabetes, coronary artery disease, stroke, atrial fibrillation, heart failure, marital status, and household income per consumption unit. Consecutive rows build on the fully adjusted model with exceptions as noted in the table.

©2020 Brunström M et al. *JAMA Network Open.*

eResults. E-values for Co-primary Outcomes

E-values with 95 % confidence intervals were calculated according to VanderWeele & Ding, Ann Intern Med. doi:10.7326/M16-2607.

For mean difference between counties during follow-up, we used the formula:

E-value = exp(0.91 x SMD) + sqrt(exp(0.91 x SMD) x (exp(0.91 x SMD) – 1)), in which

SMD = mean difference in SBP / SD for follow-up SBP = 1.05 / 16.3 = 0.064…

E-value = exp(0.91 x 0.064…) + sqrt(exp(0.91 x 0.064…) x (exp(0.91 x 0.064…) – 1))
= exp 0.058… + sqrt(exp 0.058… x (exp 0.058… - 1))
= 1.06… + sqrt(1.06… x 0.06…) = 1.31

For odds ratio for hypertension control, we used the formula:

E-value = sqrt OR + sqrt(sqrt OR x (sqrt OR – 1)) = sqrt 1.30 + sqrt(sqrt 1.30 x (sqrt 1.30 -1) = 1.54

©2020 Brunström M et al. *JAMA Network Open.*
